# Sanitize your hands and have many more benefits

**DOI:** 10.1186/2047-2994-4-S1-P164

**Published:** 2015-06-16

**Authors:** A Oliveira, S Paiva, G Faria, S Araujo, MJ Teixeira, USF Oceanos

**Affiliations:** 1 USF Oceanos, Matosinhos, Portugal

## Introduction

Hand hygiene is one of the simplest and most effective measures in reducing healthcare-associated infections. The implementation of a strategy at national level, by consensus with the proposal of the *World Health Organization*, is the most effective approach to promote the practice of hand hygiene. Portugal joined, at October 2008, the campaign *World Alliance for Patients Safety* and *Portugal’s National HealthService* has been insisting on the use of proper hand hygiene standards by health professionals and general population.

## Objectives

Evaluate the effectiveness of implementation of the campaign “*Clean Care is Safer Care*”.

## Methods

Two hundred observations of hand hygiene standards, of all professional groups, during the year 2014, in *Oceanos Health Unit*.

## Results

*Oceanos Health Unit* achieved a global improvement of hand hygiene practices, due to the involvement of all professional groups (family physician, family nurse and operational assistant). The improvement occurred in all the “five moments” of the hand hygiene, but the steepest increase happened on the fifth moment (after contact with the surrounding environment of the patient) with a difference of 62% between the pre and post campaign.

## Conclusion

Hand hygiene is one of the basic precautions with more impact on the prevention of infections. Although the 2009 data showed an undervalued adhesion to the practice of hand hygiene (46.2%), at *Oceanos Health Unit* the overall rate of compliance has increased considerably since the establishment of the strategy “*Clean Care is Safer Care*” (39 % to 67 %). The *Health Unit* is also involved in patient education as well as their caregivers in order to integrate them as partners in security.

## Disclosure of interest

None declared.

